# Antiproliferative activities of Amaryllidaceae alkaloids from *Lycoris radiata* targeting DNA topoisomerase I

**DOI:** 10.1038/srep38284

**Published:** 2016-12-06

**Authors:** Gui-Lin Chen, Yong-Qiang Tian, Jian-Lin Wu, Na Li, Ming-Quan Guo

**Affiliations:** 1 Key Laboratory of Plant Germplasm Enhancement and Specialty Agriculture, Wuhan Botanical Garden, Chinese Academy of Sciences, Wuhan 430074, China; 2 Graduate University of Chinese Academy of Sciences, Beijing 100049, China; 3 State Key Laboratory for Quality Research in Chinese Medicines, Macau University of Science and Technology, Taipa, Macau; 4 Sino-Africa Joint Research Center, Chinese Academy of Sciences, Wuhan 430074, China

## Abstract

Crude Amaryllidaceae alkaloids (AAs) extracted from *Lycoris radiata* are reported to exhibit significant anti-cancer activity. However, the specific alkaloids responsible for the pharmacodynamic activity and their targets still remain elusive. In this context, we strived to combine affinity ultrafiltration with topoisomerase I (Top I) as a target enzyme aiming to fish out specific bioactive AAs from *Lycoris radiata*. 11 AAs from *Lycoris radiata* were thus screened out, among which hippeastrine (peak 5) with the highest Enrichment factor (EF) against Top I exhibited good dose-dependent inhibition with IC_50_ at 7.25 ± 0.20 μg/mL comparable to camptothecin (positive control) at 6.72 ± 0.23 μg/mL. The molecular docking simulation further indicated the inhibitory mechanism between Top I and hippeastrine. The *in vitro* antiproliferation assays finally revealed that hippeastrine strongly inhibited the proliferation of HT-29 and Hep G2 cells in an intuitive dose-dependent manner with the IC_50_ values at 3.98 ± 0.29 μg/mL and 11.85 ± 0.20 μg/mL, respectively, and also induced significant cellular morphological changes, which further validated our screening method and the potent antineoplastic effects. Collectively, these results suggested that hippeastrine could be a very promising anticancer candidate for the therapy of cancer in the near future.

Statistically, more than 500 different kinds of Amaryllidaceae alkaloids (AAs) have been isolated from the medicinal plants of the family Amaryllidaceae^1^,^2^,^3^. Owing to the diverse pharmacological activities, such as anticancer, antimalaria, antifungal, neuroprotective effects, acetylcholinesterase and butyrylcholinesterase-inhibitory activity^4^,^5^,^6^,^7^, these alkaloids have attracted a great deal of attentions in modern medical societies. Furthermore, some AAs exhibited significant anticancer effects and were very promising in the treatment of various cancers^8^,^9^,^10^.

The AAs from *Lycoris radiata*, which has been used as a traditional Chinese medicine since long time ago, have recently drawn growing attentions since crude AAs extracts showed significant antineoplastic activities^10^. However, most of the current researches of antineoplastic activities mainly focused on either the crude total AAs or some pure compounds, the ultimately responsible bioactive components in this plant remain unclear. Recent studies showed that nearly half of the small molecule drugs are enzyme inhibitors up to now, this indicates that those small molecule drugs take effects through interacting with the target enzymes or other key biological macromolecules^11^,^12^. In addition, in the pharmaceutical industry, the binding affinity between small molecule candidates and the biomolecular targets is considered as one of the primary determinants at the early drug-discovery stage^13^.

DNA topoisomerases are nuclear enzymes and ubiquitous in prokaryotic and eukaryotic cells. By catalyzing the interconversion of topological isomers of DNA molecules in cancer cells during DNA synthesis, topoisomerases play a key part in the consecutive breakage and reunion of DNA strand^14^. Hence, topoisomerases are very attractive targets for the development of potential cancer chemotherapeutics. There usually exist two classes of DNA topoisomerases: topoisomerase I (Top I) and topoisomerase II (Top II), depending on whether they cleave the single or double strands of DNA^15^. Unlike the Top II acting on the both strands of DNA, Top I acts as the DNA-metabolizing enzyme required for the rNMPs (ribonucleoside monophosphates) -associated deletion signature without ATP hydrolysis^16^. It has been found out that topoisomerases are more liable to be attacked by the Top I inhibitors during cleavage reaction^17^. Contributed to the higher expression of Top I in tumor cells than that of normal cells, one possible mechanism is that Top I catalyzes topological interconversion of duplex DNA by reversibly relaxing and rejoining the DNA negative and positive supercoils along the phosphodiester backbone for the passage of individual DNA strands one and another^18^. As a result, the structural and functional studies on Top I have provided a reliable platform for the development of Top I inhibitors, which block the DNA synthesis and malignant cell proliferation during many pivotal cellular processes such as transcriptions, replication, chromosome condensation, and are considered as important antineoplastic chemotherapeutic agents with the mechanism of DNA interaction^19^,^20^. In clinic, Top I inhibitors have been successfully applied for the treatment of colorectal, lung and ovarian cancers nowadays^21^, such as camptothecin (CPT) families, particularly the two CPT derivatives topotecan (TPT) and irinotecan (IFL), the only two Top I inhibitors approved by the FDA for the treatments of ovarian, colorectal and lung cancer, have displayed significant anticancer effects^19^,^22^,^23^.

Inspired by the above success in developing new anticancer drugs from natural inhibitors of Top I, we selected Top I as one of the drug targets to initiate a new search for new type of natural inhibitors of Top I from *Lycoris radiata* based on our previous phytochemical and *in vitro* activity studies^24^. Thanks to the recent progress in the research and application of small molecule ligand-enzyme interaction based strategy for high throughput screening either from a combinatorial library or a complex plant extract, a number of methods have been developed to assess the ligand-enzyme binding affinity between small molecules and biological macromolecules in the last couple of decades, such as fluorescence monitoring, X-ray crystallography and calorimetric methods, magnetic resonance (NMR) and surface Plasmon resonance (SPR)^11^. However, these methods either required large amount of precious samples, or provided no or very little information about the structures of the screened inhibitors. Apart from those methods mentioned above, not only mass spectrometry (MS)-based approaches could overcome both these limitations, but also provide qualitative and quantitative information on compounds of interest with high specificity and sensitivity^24^. In this work, ultrafiltration coupled with HPLC-MS (UF-HPLC-MS) could thus be used to screen for Top I inhibitors, which could provide pivotal insights into binding properties of biomolecules with their corresponding ligands. Furthermore, the UF-HPLC-MS could also be utilized to identify numerous novel bioactive compounds online without prior tedious isolation and purification, which is very powerful for the high throughput screening (HTS) and identification of bioactive compounds from complex mixtures at early drug discovery stage^2^,^13^,^25^. Here, we presented UF-HPLC-MS based strategy to rapidly screen and identify inhibitors of Top I from the crude extracts of AAs from *Lycoris radiata*. 11 AAs were detected, and corresponding enrichment factors were then employed to evaluate the binding affinity between AAs and Top I. In this way, the best inhibitor of Top I could be fished out, and the Top I inhibition assay was then introduced to verify the potential inhibitory effectiveness of the candidate inhibitor based on its half maximal inhibitory concentration (IC_50_). Meanwhile, the molecular docking assay was carried out to simulate the interaction between Top I and the candidate inhibitor of interest. At last, antiproliferation assays on human colon carcinoma cells (HT-29) and hepatocellular liver carcinoma cells (Hep G2) *in vitro* were conducted to further validate our screening results and the potential antineoplastic effects. For the first time, new Top I inhibitors from *Lycoris radiata* were systematically screened and identified, and compound 5 was, first and foremost, reported to exhibit potent antineoplastic activity, which is comparable with the well known anticancer drug like camptothecin. To some extent, our present work could also provide very important clues for the future anti-cancer mechanisms of action regarding compound 5 from AAs.

## Results and Discussion

### Ultrafiltration of compounds bound to Top I

**Figure 1:**
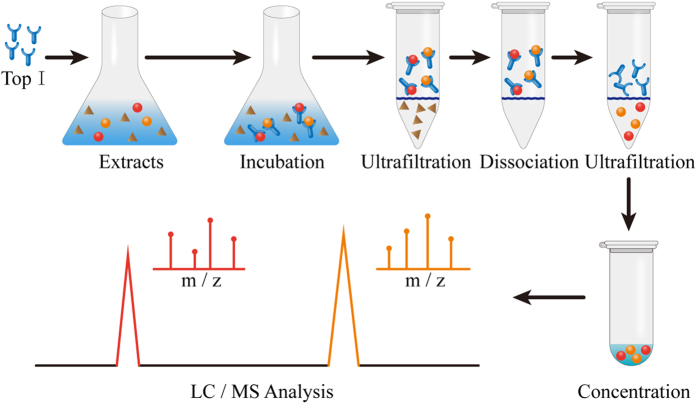
Schematic diagram of UF-HPLC/MS assay to screen for Top I inhibitors. The principle of the assay usually involves three steps, including incubation, ultrafiltration and identification.

In sharp contrast to the traditional phytochemical study on medicinal plants, which often requires labor-intensive and time-consuming multiple-step procedures for the isolation of pure compounds from medicinal plants, and subsequent bioactivity tests, bioaffinity ultrafiltration method based on the interactions between small molecular ligands and the active sites of enzymes^25^, is much more effective. Meanwhile, bioaffinity ultrafiltration combining with HPLC-MS could further offer vital insights into chemical structures of bioactive candidates of interests, and ligand-receptor binding properties^13^,^26^. Generally speaking, the principle of UF-HPLC-MS assay usually involves three steps, including incubation, ultrafiltration and identification, and the proposed scheme is illustrated in Fig. 1. Briefly, in the assay, after the incubation of the complex mixtures of compounds from a crude plant extract with potential target enzymes, the bioaffinity ultrafiltration separates the ligand-receptor complexes from the unbound compounds, later the bound ligands released from the complexes could be subsequently identified and quantified by HPLC-MS/MS analysis.

**Figure 2:**
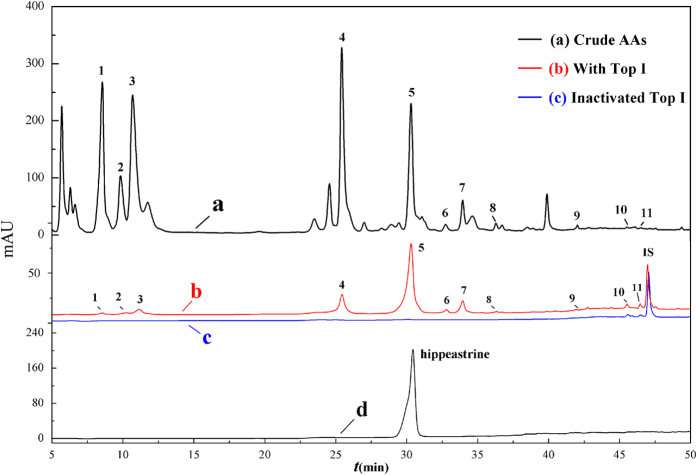
HPLC chromatograms of the chemical constituents in crude AAs obtained by ultrafiltration (at 232 nm). The black solid line (a) represents HPLC profiles of the crude AAs without ultrafiltration. The red line (b) and blue line (c) represent the crude AAs with activated and inactivated Top I, respectively. Nuciferin was used as the internal standard (IS). Peak 5 was further confirmed with the standard compound of hippeastrine under the same conditions (d).

Obviously, the chromatogram of AAs shows distinct differences before and after incubation with Top I as shown in Fig. 2. 11 components in the AAs exerted specific binding affinity to Top I, and those components in AAs incubated with Top I gave bigger peak areas than those of the inactivated control group, which were considered as potential ligands of Top I. Meanwhile, the relative amounts of the 11 peaks from both activated and inactivated group were calculated and shown in Table 1. It’s interesting that the relative amount of 11 components incubated with activated Top I are significantly higher than those with inactivated controls, and the amount of components 1–9 is barely detected or negligible when incubated with the inactivated Top I.

Based on the variations of the peak areas before and after incubation with Top I, the enrichment factor is defined as the degree of affinity binding between the ligands and the enzyme. The enrichment factor (EF) was calculated as follows: EF = (A_T_ − A_C_)/A_0_ × 100%, where A_T_, A_C_, A_0_ represent the peak areas obtained in the experiment involving incubation with activated, inactivated and without Top I in Fig. 2 12, respectively. Among those chemical constituents from AAs, the unique EF is used to assess specific and nonspecific binding of each compound to Top I, and the characteristic bioactivities such as antineoplastic activity in this study could thus be implied. It showed clearly in Table 1 that peak 5 possess the greatest degree of binding affinity (49.3%), followed by 7 (24.2%), 4 (12.7%) and 6 (11.1%). As expected, the EFs for each compound were different from each other. It is noteworthy that even those components with much higher abundances, like peaks 1, 3, and 4 in Fig. 2 exerted a relative lower binding affinity to Top I, and the discrepant EFs may indicate that the distinguished competitive relationships among these bioactive components bound to Top I exist.

### Identification of Top I inhibitors from crude AAs

After incubation with Top I and ultrafiltration affinity screening, 11 components in the AAs in Fig. 2 exerted specific bindings. The ESI-MS/MS analysis of these 11 peaks was conducted in the positive ion mode, and their retention times (Rt), calculated molecular masses, and MS/MS data are shown in Table 1, respectively.

Based on the comparisons of MS/MS data with the reported literatures, peaks 1, 2 and 3 were identified as lycorine, lycoramine and galanthamine^2^,^24^, respectively. In regard to peak 4 ([M + H]^+^ at m/z 332), the fragment at m/z 300, 282 and 264 were obtained by the corresponding neutral loss of CH_4_O, H_2_O and CH_6_O_2_, respectively. Due to the RDA (retro Diels-Alder reaction) cleavage, fragments at m/z 213 and 225 were derived from the loss of C_3_HN and C_2_HN. Compared with the MS/MS data reported, peak 4 was identified as ambelline^2^. Interestingly, peaks 5, 6, 7, 8 and 9 shared a same chemical skeleton, and were identified as homolycorine type AAs. The two peaks at 5 ([M + H]^+^ at m/z 316) and 6 ([M + H]^+^ at m/z 334) were identified as hippeastrine, and 2α-hydroxy-6-O-methyloduline according to the previous study, respectively^27^. For peak 5, the characteristic fragments at m/z 191 and 126 were yielded due to the RDA rearrangements. The fragments at 298, 280 and 239 were obtained because of the loss of H_2_O, 2H_2_O and C_3_H_11_NO. Furthermore, peak 5 was confirmed by comparing the retention time and the MS/MS spectra with the corresponding standard (Fig. 2d). As for peak 10 ([M + H]^+^ at m/z 346), the same abundant fragment ions at m/z 211, 181 and 168 as those of of haemanthamine indicated it shared the similar chemical structure^2^. In addition, the fragment ion at m/z 288 was yielded due to the loss of C_2_H_4_NO through the RDA cleavage, and other fragments at m/z 241, 239, 211, 183 were obtained due to the loss of its corresponding substituents, peak 10, accordingly, was assigned as (+)-3α-hydroxy-6β- acetylbulbispermine^28^. So far, the structures of these ten AAs were successfully identified.

### Top I inhibition assay *in vitro*

During cell proliferation, Top I involves in the controlling and modification of topological heterogeneous states of DNA molecules. Because Top I is highly expressed in cancer cells, inhibiting Top I could rapidly suppress the proliferation of cancer cells^18^,^29^,^30^. After ultrafiltration with the Top I, peak 5 (hippeastrine) showed the highest EF value of 49.3%. In order to verify the effectiveness of the UF-LC/MS based method and inhibitory capacity of hippeastrine against Top I, the IC_50_ value was determined using an *in vitro* enzymatic inhibition assay.

**Figure 3:**
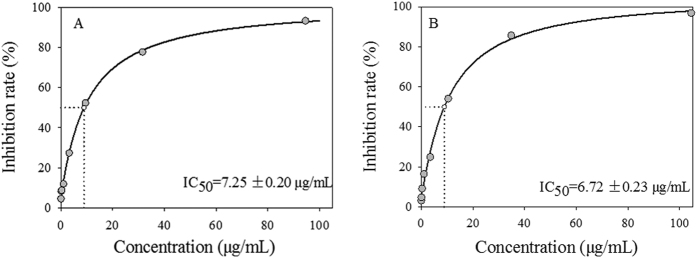
The half-maximal inhibitory concentrations (IC50) of hippeastrine (**A**) and camptothecin (**B**) on Top I *in vitro*. The two compounds showed similar dose-dependent manners with the IC_50_ at 7.25 ± 0.20 μg/mL and 6.72 ± 0.23 μg/mL, respectively.

The IC_50_ value of the hippeastrine was evaluated in a concentration range of 0.03–100 μg/mL. Camptothecin, the first small molecule targeting Top I for the treatment of advanced digestive carcinoma in clinical^23^,^31^, which stabilizes the DNA cleavable complex to block the transient breaking and rejoining of DNA^17^,^32^,^33^ and has been used as the Top I poison for the treatment of many digestive solid tumors widely^34^, was served as the positive control. As shown in Fig. 3, hippeastrine and camptothecin exhibited inhibitory activity on Top I in a similar dose-dependent manner with the IC_50_ values at 7.25 ± 0.20 μg/mL and 6.72 ± 0.23 μg/mL, respectively, which clearly implied that hippeastrine was found to be comparable with the well known anticancer drug camptothecin in terms of IC_50_. Other tests on camptothecin against Top I also showed similar inhibitory activity levels with the IC_50_ values at 8.71 μg/mL^35^ or 8.53 μg/mL^36^, which could further approve our finding in this work. Hence, our result confirmed that hippeastrine could be a potential Top I inhibitor as a very promising anticancer drug candidate, which is in good consistent with the enrichment factors based on UF-LC/MS assay and provides a good validation for its effectiveness. Since Top I relaxes supercoils by reversibly nicking duplex DNA to control DNA replication^16^, hypothesis is that hippeastrine could reversibly block Top I -mediated cleavage of DNA complex, finally causing the DNA strand breaks and activation of apoptosis^11^.

### Molecular docking

**Figure 4:**
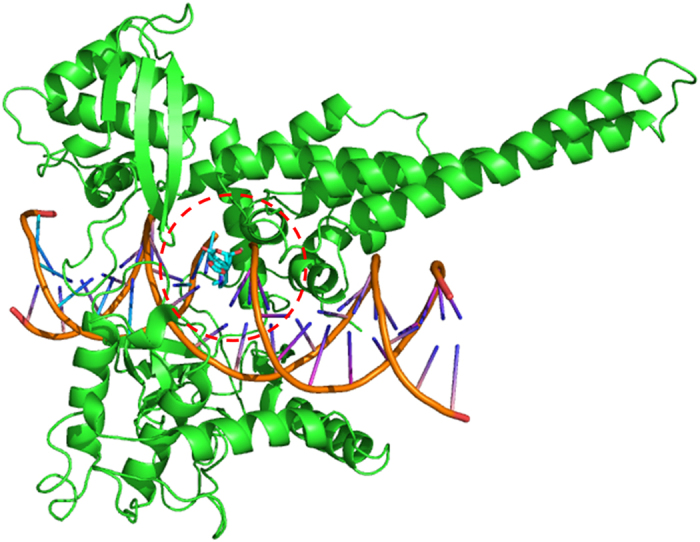
Molecular docking simulation between hippeastrine and Top I-DNA complex. Ribbon (green) represents the Top I (PDB ID: 1T8I), and the embedded hippeastrine (purple) shows covalent binding with a 22 bp DNA.

Due to the distinct inhibitory activity of hippeastrine on Top I *in vitro*, the molecular docking assay was carried out consequently to rationalize its activity on Top I. After the energy minimization by the MMFF94× force field, the ligand (9.9 Å, length) possessing the lowest energy was used for the molecular docking simulation. The crystal structures of Top I (PDB ID: 1T8I) and hippeastrine covalently combined with a 22 bp (base pair) DNA (Fig. 4). It showed the free binding energy of −6.9 kcal/M between the hippeastrine and Top I from the docking processes. Meanwhile, it further revealed that the intermolecular interactions of hydrophobic effect and Vad der Waals force were the major driven forces between the receptor-drug conjugate. Hence, the non-covalent binding has proven to mediate the complexes. It was also conjectured in Fig. 4 that the small molecular ligand (hippeastrine) firstly entered into the active hydrophobic pocket formed between the DNA and Top I due to the hydrophobic effect, and then modulated the receptor protein of Top I.

**Figure 5:**
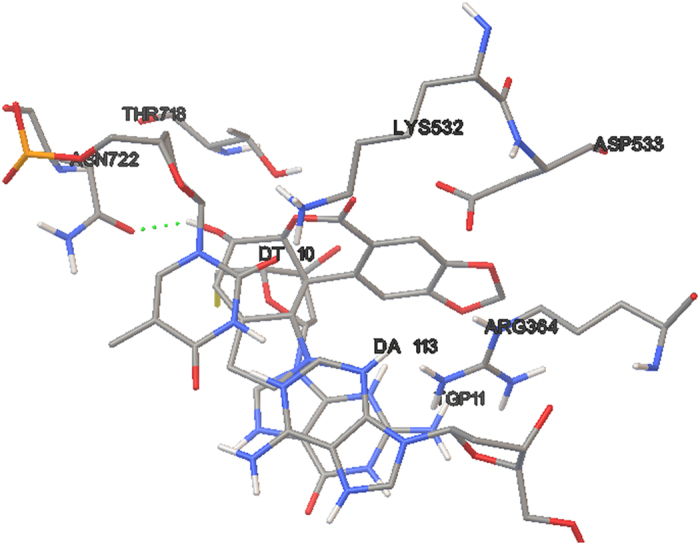
The predicted active binding sites of hippeastrine with Top I. The dotted line (green) represents the hydrogen-bonding interactions.

As a result, hippeastrine was observed to interact with the active site residues of Top I, namely, Asp 533, Lys 532, Arg 364, Thr 718 and Asn 722, where Asp 533 and Arg 364 were required for camptothecin to bind Top I as reported, and thereafter reacted on the binding sites of DA10, DA113 and TGP11 in DNA (Fig. 4). Moreover, the H-bonds (hydrogen bonds) formed between the hydroxyl group of hippeastrine and the residue Asn722 of Top I strengthened the binding ability (Fig. 5), which indicated the formation of H-bonds played a key role in the binding between hippeastrine and Top I. Considering the molecular docking results above, it is assumed that hippeastrine truly reacted on the amino acid residues and further stabilized the Top I-DNA cleavage complex to competitively inhibit the activity of Top I.

### Antiproliferation assays and determination of the IC_50_ on human cancer cell lines of HT-29 and Hep G2

According to the previous study, the majority of bioactive alkaloids exhibiting higher potential anti-Hep G2 activity from the *Lycoris radiata* are mainly lycorine, galanthamine and homolycorine types^24^. The bulbs of *lycoris radiata* have been extensively used as a traditional Chinese folk medicine for thousands of years, and eventually the phytochemical investigations have led to the isolation of various types of alkaloids with diverse biological activities. For example, lycorine could dramatically suppress the growth of RAW 264.7 and leukemia cells^37^,^38^. Homolycorine, which also belonged to the lycorenine type, showed promising antiproliferative activities against HeLa (human cervical adenocarcinoma), Vero (monkey kidney epithelium) and Jurakat (human T-cell leukemia) cell lines^39^. Galanthamine and lycoramine were also reported to exhibit acetylcholineaterase (ACHE) inhibitory activity and neuroprotective effect for the treatment of Alzheimer’s disease^1^,^40^. It is well known that many AAs isolated from Amaryllidaceae plants, such as narciprimine, arolycoricidine and distichamine, are the important secondary metabolites used for the treatment of cancer. Unfortunately, none of such a pure compound has been applied for the clinical trials to date^1^,^41^.

As a matter of fact, for three out of the four potential bioactive AAs of higher EFs are homolycorine type on the basis of the UF-HPLC-MS assay above. At the same time, the inhibitory assay *in vitro* also showed that the peak 5, which was deduced by its MS/MS, and further confirmed with the standard compound namely hippeastrine (Fig. 2), with the highest EF value of 49.3%, exhibited a good dose-dependent inhibitory effect against Top I with IC_50_ at 7.25 ± 0.20 μg/mL. To further validate our screening method and the antineoplastic effects, the *in vitro* antiproliferation activities of hippeastrine on human cancer cell lines of HT-29 and Hep G2 were evaluated at a concentration range of 0.37–30.0 μg/mL in this test. Here, 5-FU was applied as another positive control especially for the HT-29, which has been commonly used for the treatment of colorectal cancer (CRC)^42^,^43^. Additionally, 5-FU, associated with several targeted therapies, such as anti-VEGF or anti-EGFR1 monoclonal antibodies, has been the backbone for the treatment of digestive solid cancer patients^43^,^44^. The degrees of antiproliferation against human carcinoma cell lines resulting from treatments were evaluated by the MTT assay, and the growth inhibitory rate was expressed as the percentage of the total cells compared with the negative control after 72 hours treatment. Studies showed that a number of Amaryllidaceae alkaloids and their derivatives exhibited remarkable antiproliferative activities^8^,^9^,^10^,^41^,^45^. Our results in Table 2 also displays that hippeastrine, [2] benzopyrano [3,4] indole skeleton based lycorenine-type alkaloids, exhibited distinct dose-dependent antiproliferative activities against HT-29 and Hep G2 cells with the IC_50_ values at 3.98 ± 0.29 μg/mL and 11.85 ± 0.20 μg/mL, as compared to that of camptothecin at 1.47 ± 0.07 μg/mL and 3.17 ± 0.56 μg/mL, 5-FU at 2.92 ± 0.48 μg/mL, respectively. Notably, hippeastrine is more sensitive against HT-29 with a comparable IC_50_ to that of 5-FU. It has been known that Top I is highly expressed in colorectal cancers, and repeated exposure of camptothecin to colorectal cancer xenografts could lead to downregulation of Top I levels^30^,^46^. As detailed above, the significant antiproliferative effect against HT-29 in the experiment further confirm the hypothesis that the highly expressed Top I could predict response to hippeastrine. Together with the *in vitro* Top I inhibitory assay in this regard, it is proposed that hippeastrine exhibits prominent antiproliferative effects through disrupting topological interconversion of duplex DNA then further blocking DNA synthesis. Accordingly, hippeastrine could be a promising anticancer candidate. In addition, structure-activity relationship analysis revealed that lycorine, the precursor of hippeastrine, displayed antiproliferative activities against six distinct cancer cell lines through the cytochrome c-mediated and caspase-dependent pathway and was considered as a good apoptosis inducer^27^,^39^,^45^. Further study suggested the phenanthridone skeleton, a common minimal structural feature in alkaloids of the Amaryllidaceae family, such as pancretistatin and their congeners, may be responsible for these cell specific anti-cancer agents^47^. Consistent with the above results, several alkaloids including hippeastrine from the *Narcissus L*., another plant in Amaryllidaceae family, showed antiproliferative activities on Hela, Vero and Jurkat cell lines, which induced the nuclear morphological changes associated with the possible mechanism of apoptosis^10^,^39^.

**Figure 6:**
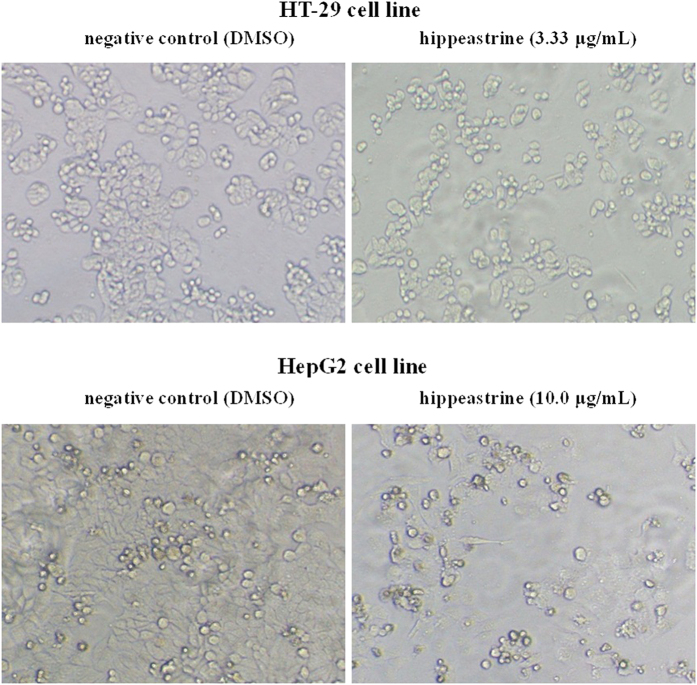
Morphological illustrations of HT-29 and Hep G2 cell populations treated for 72 h with hippeastrine (100×). The illustrations demonstrate the strong growth inhibitory activity of hippeastrine using phase-contrast microscopy. The concentrations of 3.33 μg/mL and 10.0 μg/mL here are very near to the IC_50_ values of HT-29 and HepG2 acquired in the MTT assay *in vitro*, respectively.

After treated with hippeastrine for 72 h, cell populations and morphological changes of HT-29 and Hep G2 were observed with a phase-contrast microscopy. The concentrations of 3.33 μg/mL and 10.0 μg/mL chosen here approximately equal to the 50% inhibitory rates of HT-29 and Hep G2. Significant reduction of viable cells caused by the drug treatment was observed as shown in Fig. 6. At the same time, the numbers of viable cells exerted a distinct dose-dependent manner in the other groups, which were also in accordance with the previous MTT results. Other morphological changes also included cell shrinkage, decreased intercellular adhesion, scattering and expanded intercellular spaces. Whereas the negative control cells maintained the normal epithelial morphology. In the early stages of apoptosis, many morphological changes of apoptotic features such as cell shrinkage, membrane blebbing and so on occurred commonly in HT-29 cells^48^,^49^. Intervention with hippeastrine, therefore, caused significant pharmacodynamic effects on the cellular morphology of those cancer cells, which were very similar to the results of the previous study^39^.

## Conclusion

In this study, a UF-HPLC-MS method was developed to screen Top I inhibitors from crude alkaloids in *Lycoris radiata*. 11 alkaloids showed potential inhibitory activity, 10 of which were identified according to their MS/MS spectra and fragmentation pathways. The enzymatic inhibition assay against Top I *in vitro* was carried out, and the results showed that the compound 5, namely hippeastrine, with the highest EF value of 49.3%, exhibited a good dose-dependent inhibitory effect against Top I with IC_50_ at 7.25 ± 0.20 μg/mL, as compared to the positive control (camptothecin) at 6.72 ± 0.23 μg/mL. Furthermore, the molecular docking simulation indicated that hippeastrine interacted with the amino acid residues of Top I through H-bonds and further stabilized the Top I-DNA cleavage complex to competitively inhibit the activity of Top I. Finally, the antiproliferation assay on HT-29 and Hep G2 *in vitro* revealed that hippeastrine strongly inhibited the growth of cancer cell lines in an intuitive dose-dependent manner with the IC_50_ values at 3.98 ± 0.29 μg/mL and 11.85 ± 0.20 μg/mL, respectively, which further validated our screening method and the potential antineoplastic effects. Cell populations and morphology of cancer cells also changed dramatically when treated with hippeastrine using a phase-contrast microscopy. To conclude, our results strongly suggested that hippeastrine could be a potential anticancer candidate for future cancer therapeutics. Further studies should focus on the possible antiproliferative molecular mechanisms induced by hippeastrine.

## Methods

### Materials, chemicals and reagents

DNA topoisomerase I (*E. coli*) was purchased from New England Biolabs (NEB, Ipswich, Massachusetts, USA). The hippeastrine was provided by Accurate Chemical & Scientific Corp. (Westbury, New York, USA). The centrifugal ultrafiltration filters (YM-30, 30 kDa) were provided by Millipore Co. Ltd (Bedford, MA, USA). The HT-29 cell line was purchased from China Center for Type Culture Collection (CCTCC, Wuhan, China). Acetonitrile (ACN) and ammonium acetate (AA) were purchased from TEDIA Company INC (Fairfield, Ohio, USA). Water for ultrafiltration and HPLC-MS analysis was prepared with EPED (Nanjing Yeap Esselte Technology Development Co. Ltd, Nanjing, China). All other chemicals and solvents were of analytical grade.

Fresh bulbs of *Lycoris radiata (L. radiata*) were collected from Wuhan Botanical Garden, which were kindly authenticated and identified by the taxonomist (Dr. Guangwan Hu) of Key Laboratory of Plant Germplasm Enhancement and Specialty Agriculture (Wuhan Botanical Garden), Chinese Academy of Sciences. A voucher specimen (No. 0019) was deposited in the herbarium of the Key Laboratory.

### Sample preparation and ultrafiltration screening

The fresh raw bulbs were sliced into pieces, dried in the oven then ground into powder. Later, the powdered sample (100.0 g) was accurately weighted and followed by ultrasonic extraction, and the crude AAs were finally prepared as reported in our previous study^24^.

The ultrafiltration screening procedure was carried out according to the previous study with some modifications^50^. Briefly, an aliquot of 100 μL AAs sample solution (2.0 mg/mL) and 10 μL topoisomerase I (0.5 U/μL) were added successively into a 0.2 mL EP tube and set as the experimental group. At the same time, the inactivated topoisomerase I solution (boiled for 10 min in water bath) was conducted as the control in a similar manner. Then the mixtures were incubated at 37 °C for 30 min. After that, the incubation solutions were transferred into a 30 kDa molecule weight cut-off centrifugal ultrafiltration filter (YM-30) and centrifuged at 10,000 rpm for 10 min at room temperature. The filtrates were washed 3 times by centrifugation with 200 μL of NE Buffer (pH 7.9, 25 °C) to remove the unbound compounds. Then the ligands with specific binding to Top I were dissociated from the complexes by adding 200 μL ACN/water (90:10, v/v), and let stand for 10 min, and then centrifuged at 10,000 rpm for 10 min. The dissociation process was repeated 2 times. Finally, the filtrates were lyophilized with a centrifugal evaporator, reconstituted in 50 μL 90% aqueous ACN (containing 5 μg/mL nuciferine as the internal standard) and directly analyzed by the HPLC-ESI-MS/MS system.

### HPLC-ESI-MS/MS analysis

The crude AAs dissolved in methanol and the two tubes of filtrates mentioned above were directly analyzed by HPLC-ESI-MS/MS. The HPLC-ESI-MS/MS analysis was performed with a TSQ Quantum Access MAX mass spectrometer (Thermo Fisher Scientific, San Jose, CA, USA) coupled with a Thermo Accela 600 HPLC system. The chromatographic separation was carried out on a Phenomenex ODS column (150 × 2.00 mm, 5 μm) at a flow rate of 0.2 mL/min. The column temperature was set at 30 °C. An aliquot of 10 μL sample solution was injected into the HPLC system, and a binary gradient LC conditions were: solvent A (40 mM ammonium acetate), and solvent B (ACN). The HPLC elution method was as follows: 0–15 min, 5% (B); 15–17 min, 5–10% (B); 17–20 min, 10% (B); 20–30 min, 10–18% (B); 30–55 min, 18–68% (B). The Online UV chromatograms were acquired at the wavelength of 232 nm.

For the ESI-MS/MS analysis, the mass spectrometer operated in the positive ion mode, and the optimized instrument conditions of MS were set as follows: spray voltage, 3.0 kV; capillary temperature, 250 °C; vaporizer temperature, 350 °C; cone voltage, 40.0 V; Sheath gas pressure, 40 psi; Aux gas pressure, 10 psi. Collision energies for the MS/MS analysis ranged from 30–45 eV in accordance with the mass of the precursor ion. Nitrogen (N_2_) was served as the cone and desolation gas, and helium (He) was used as the collision gas. Mass spectrometry data were acquired n full-scan mode for m/z in the range from 200 to 1,000. All data acquisition and analysis was performed in the Thermo Xcalibur ChemStation (Thermo Fisher Scientific, Waltham, MA, USA).

### Quantitative and qualitative Analysis of AAs

The relative quantitation of active ingredients screened by ultrafiltration were calculated in accordance with the peak areas from the HPLC chromatography against nuciferine. For further illustration of those chemical structures, the identification and characterization of corresponding peaks was deduced from their MS/MS spectra, and in comparison with the relevant reference standards, and fragment pathways reported previously.

### Top I inhibition assay *in vitro*

DNA Top I inhibition assay was conducted according to the methods described previously with some modification^14^,^51^. The reaction buffer included 50 mM Kac, 20 mM Tris-Ac, 10 mM Mg(Ac)_2_, 1 mM dithiothreitol (DTT) and 0.01% bovine serum albumin (BSA). DNA Top I (2.0 U, *E. coli*) and the test compound at the indicated concentrations of 0.032–31.53 μg/mL (0.1–100 μM) were placed into a 96- well plates in a final volume of 100 μL. The reaction mixtures were incubated at 37 °C for 30 min, and then terminated by the addition of 20 μL stop solution (5% SDS and 50 mM EDTA). The absorbance of the reaction mixtures was measured at 510 and 590 nm with a Tecan plate reader (Infinite M1000, Switzerland). Camptothecin, a well known Top I inhibitor, was used as the positive control. Each sample solution was implemented in triplicate, and the results were expressed as means ± SD (standard deviation). IC_50_ values were executed by nonlinear regression analysis and sigmoidal dose response curves were obtained using SigmaPlot, version 12.5.

### Molecular docking assay

The molecular docking simulations between Top I and hippeastrine was carried out using the Genetic Algorithm of AutoDock 4.2 software^52^. Briefly, the 3D structure of hippeastrine was established with MOE Molecule Builder tool, and then its energy minimization was executed by the MMFF94× force field. The water molecules were removed, and after that the hydrogen atoms were added. The centroid coordinate (the red circle, Fig. 4) of the receptor-drug crystal conjugate was served as the docking site. Docking calculations were manipulated using 2.5 × 10^7^ energy evaluations and the default parameters (runs 30). Meanwhile, the rotatable bonds of hippeastrine were specified with the AutoDock Tools. Finally, a grid map of 60 × 60 × 60 nearby the docking site was constructed to calculate the energy scoring using the Autogrid.

### Antiproliferation assays and determination of IC_50_ on HT-29 and Hep G2 cells

The *in vitro* antiproliferation activities of hippeastrine on HT-29 and Hep G2 were evaluated by MTT (3-(4,5-dimethyl-2-thiazolyl)-2,5-diphenyl-2-H-tetrazolium bromide) assay in a concentration range of 0.37–30.0 μg/mL. In brief, 35,000 cells per well were seeded into 96-well plate of DMEM (Dulbecco’s Modified Eagle Medium), supplemented with 10% fetal bovine serum (FBS), 1% penicillin-streptomycin and incubated in a humidified atmosphere containing 5% CO_2_ at 37 °C for 24 h. The hippeastrine was dissolved in dimethyl sulfoxide (DMSO), which was conducted as the blank control, and then diluted into the relevant final concentrations with the medium. After 72 hours of drug treatment, 20 μL of MTT solution (5 mg/mL) was added into each well and the plate was incubated for another 4 h. The optical density (OD) value of each well was measured at 490 nm using a Tecan plate reader. Camptothecin and 5-FU served as the positive controls. The IC_50_ value was defined as the concentration that caused a 50% reduction of absorbance at 490 nm in treated cells compared with the untreated controls. Each sample solution was carried out in triplicate, and the results were expressed as means ± SD.

## Additional Information

**How to cite this article**: Chen, G.-L. *et al*. Antiproliferative activities of Amaryllidaceae alkaloids from *Lycoris radiata* targeting DNA topoisomerase I. *Sci. Rep.* **6**, 38284; doi: 10.1038/srep38284 (2016).

**Publisher's note:** Springer Nature remains neutral with regard to jurisdictional claims in published maps and institutional affiliations.

## Figures and Tables

**Table 1:** The relative amounts and ultrafiltration HPLC-ESI-MS/MS spectra of the bioactive compounds bound to Top I from AAs.

Peak No.	Rt (min)	[M + H]^+^	Relative amount (μg/mL)		EFs (%)	MS/MS data
			AAs -T	AAs -C		
1	8.5	288	0.15	0	0.4	270, 252, 222, 177, 147, 119
2	10.2	290	0.14	0	1.3	272, 233, 215, 189
3	11.1	288	0.78	0	2.3	270, 231, 225, 213, 198
4	25.4	332	4.96	0	12.7	300, 282, 264, 234, 225, 213, 199, 169
5	30.3	316	15.93	0	49.3	298, 280, 273, 239, 191, 126, 96
6	32.8	334	0.28	0	11.1	316, 298, 270, 267, 255, 238, 173
7	33.9	316	1.62	0	24.2	298, 280, 267, 239, 237, 207, 191, 176
8	36.3	332	0.08	0	4.1	300, 282, 271, 257, 243, 191
9	42.7	332	0.05	0	2.6	314, 300, 282, 271, 257, 191, 181, 175
10	45.5	346	0.28	0.14	8.3	288, 271, 241, 239, 211, 193, 183, 181, 168
11	46.4	556	0.22	0.10	6.1	282, 267, 266, 251, 220

(Note: 5 μg/mL nucifucin as the internal standard). AAs-T and AAs-C represent the experiments of AAs with activated and inactivated Top I, respectively.

**Table 2:** The half-maximal inhibitory concentrations (IC_50_ values) of hippeastrine (Peak 5) on human cancer cell lines of HT-29 (colon carcinoma) and HepG2 (liver cancer).

Compounds	IC_50_ (μg/mL)	
	HT-29	HepG2
Hippeastrine (Peak 5)	3.98 ± 0.29	11.85 ± 0.20
Camptothecin	1.47 ± 0.07	3.17 ± 0.56
5-Fu	2.92 ± 0.48	—

Camptothecin and 5-FU were served as the positive controls.
